# COVID-19: Recent Insight in Genomic Feature, Pathogenesis, Immunological Biomarkers, Treatment Options and Clinical Updates on SARS-CoV-2

**DOI:** 10.2174/0113892029291098240129113500

**Published:** 2024-02-07

**Authors:** Rohitas Deshmukh, Ranjit Kumar Harwansh, Akash Garg, Sakshi Mishra, Rutvi Agrawal, Rajendra Jangde

**Affiliations:** 1 Department of Pharmaceutics, Institute of Pharmaceutical Research, GLA University, Mathura, 281406, Uttar Pradesh, India;; 2 Department of Pharmaceutics, Rajiv Academy for Pharmacy, NH-2, Mathura, Delhi Road, Chhatikara, 281001, Uttar Pradesh, India;; 3 Institute of Pharmacy, Pt. Ravishankar Shukla University, Raipur, Chhattisgarh, 492010, India

**Keywords:** SARS-CoV-2, COVID-19, coronavirus, biomarkers, omicron, delta variant

## Abstract

SARS-CoV-2 is a highly contagious and transmissible viral infection that first emerged in 2019 and since then has sparked an epidemic of severe respiratory problems identified as “coronavirus disease 2019” (COVID-19) that causes a hazard to human life and safety. The virus developed mainly from bats. The current epidemic has presented a significant warning to life across the world by showing mutation. There are different tests available for testing Coronavirus, and RT-PCR is the best, giving more accurate results, but it is also time-consuming. There are different options available for treating n-CoV-19, which include medications such as Remdesivir, corticosteroids, plasma therapy, Dexamethasone therapy, *etc*. The development of vaccines such as BNT126b2, ChAdOX1, mRNA-1273 and BBIBP-CorV has provided great relief in dealing with the virus as they decreased the mortality rate. BNT126b2 and ChAdOX1 are two n-CoV vaccines found to be most effective in controlling the spread of infection. In the future, nanotechnology-based vaccines and immune engineering techniques can be helpful for further research on Coronavirus and treatment of this deadly virus. The existing knowledge about the existence of SARS-CoV-2, along with its variants, is summarized in this review. This review, based on recently published findings, presents the core genetics of COVID-19, including heritable characteristics, pathogenesis, immunological biomarkers, treatment options and clinical updates on the virus, along with patents.

## 1 INTRODUCTION

Coronaviruses are a type of infectious pathogen that can induce moderate to extreme respiratory problems. SARS-CoV-2, the novel coronavirus, was originally introduced in the Hubei province of China in December 2019 and caused an unusual viral pneumonia outbreak. Since then, it has rapidly transmitted across the world and has vigorously crossed SARS-CoV and MERS-CoV in the sense that more people contract the virus and the geographic distribution of epidemic zones [1, 2]. COVID-19 is an acute viral respiratory illness-induced variation of Coronavirus dubbed SARS-CoV-2 that has killed more than 3 million populations worldwide as of May 1, 2021. Although most people recover on their own with mild fever and discomfort, a small percentage of them may experience more severe symptoms and develop life-threatening conditions, such as respiratory failure (ARDS), that could cause systemic multi-organ failure. Since SARS-CoV-2 is a novel virus with few antiviral drugs that are being repurposed for COVID-19 management (*e.g*., Remdesivir), an enhanced comprehension of the virus pathobiology is needed to develop prophylactic and/or therapeutic strategies [3, 4]. The illustration of main cell signalling is shown in Fig. (**1**) [5].

Insights into the life cycle of SARS-CoV-2, its pathophysiology, and rationalized therapies for COVID-19 clinical complications.

## 2 GENOMIC FEATURES OF SARS-COV-2

CoV genomes are between 26 and 32 kilobases (kb) [6]. Specifically, a genomic examination of another RNA infection strain initially called Wuhan-Hu-1 uncovered that a specific variant of SARS-CoV-2 is nearly 30 kb in size [7]. The underlying and non-primary proteins are coded for by viral RNA. Inside the 3' finish of the RNA genome, primary proteins are framed. Non-underlying proteins (nsps), which are required for replication, are encoded by 66% of the genome [6, 8]. The previously mentioned genomic material is first utilized as a layout for the union of the polyprotein, which encodes for NSPS, which is coordinated in twofold film vesicles. These sub-genomic courier RNAs all have a similar 70-90 nucleotide pioneer grouping at their 5' closes and a similar 3' closes. Record successions are found between open understanding casings, record end, and the procurement of a pioneer RNA (ORFs). These particular less-strand sgRNAs fill in as layouts for sub-genomic mRNA arrangement [9, 10]. The genomic material for SARS-CoV-2 is depicted in Fig. (**2**) [11]. Aside from gamma CoV, which needs nsp1, 66% of viral RNA is contained in the main ORFs, which encode 16 NSPS [12].

By this, SARS-CoV-2 proteins are practically like SARS-CoVs, with minor varieties. Another examination found that changes in both nsp2 and nsp3 are required for the infection's separation system [13, 14]. At last, as per another Chinese examination researching the SARS-CoV-2 genotypes of people from various regions, the infection has an expanded change rate in numerous individuals [15].

## 3 VIRAL INTERNAL COMPONENTS AND THEIR UNIQUE CHARACTERS

The name coronavirus, derived from the Latin word corona, meaning crown, was given to the virus because of its spherical shape and spike projections, which made the virus seem under an electron microscope like a royal crown [16]. The primary structural proteins that make up the structure of Coronavirus are spike (S), membrane (M), envelope (E) & nucleocapsid (N) proteins [17]. In certain cases, beta coronaviruses also contain hemagglutinin-esterase (HE) protein [18]. Coronaviruses are enveloped viruses, with the envelope being a lipid bilayer derived from the host cell membrane. N protein, which is found in the centre of a viral particle and integrates with the viral RNA to create the nucleocapsid [19], is different from S, M, & E proteins, which are all encased within the viral envelope [8]. Viral passage into host cells is mediated by the highly glycosylated S protein, which forms homotrimeric spikes on the outer layer of the viral particle [8, 20].

## 4 PATHOGENESIS OF NOVEL CORONAVIRUS

Coronavirus patients have some clinical highlights that have been set up. Neurotic interstitial pneumonia and conspicuous aspiratory oedema, just as protein exudation and gentle incendiary cell invasion, were discovered to be early obsessive changes incited by SARS-CoV-2 pneumonia [21]. The infection was found in higher plenitude in alveolar epithelial cells, though protein union capacity in vessel dividers and interstitial regions was low [15]. Platelet accumulation and fibrin affidavit can cooperate to advance the development of thrombi in the lungs' fringe little vessels [22]. Sings of harm to different organs as well as cycles, then again, require more complete examination discoveries [23].

## 5 TRANSMISSION AND CAUSES OF NOVEL VIRAL INFECTIONS IN HOST

Multiple cases of unexplained respiratory infections, which first occurred in China, were believed to have occurred by first-hand contact with a lump of marine meat. The primary mechanism is supposed to be zoonotic transmission. Bats have been identified as a probable vector for novel Coronavirus in many reports. The transmission of the Coronavirus is shown in Fig. (**3**) [24]. Regardless, it is unclear if SARS-CoV-2 got its start in the seafood industry. As a result, it was observed that social experiences could also be a source of virus transmission [4].

As per clinical records from case arrangements around there and information from nearby CDCs, 72.3 percent of non-inhabitants of Wuhan had recently had contact with Wuhan occupants, and 31.3 percent had, as of late, went to this city [25]. Moreover, it was recorded that the infection could hatch for 3 to 7 days and as long as about 14 days, with the longest time from disease to symptomatology being 12.5 days [26]. Fomites are additionally thought to be the essential wellspring of irresistible particles; however, this is easy to refute [27, 28].

## 6 KEY TARGETS OF SARS-COV-2

As per epidemiological exploration, 77.8% of Coronavirus patients are between the ages of 30 and 69, with the biggest proportion in the 50- to 60-year-mature age group, while the disease occurrence in youngsters is generally low [29]. SARS-CoV-2 is thought to have a more remarkable spreadability than SARS-CoV. The essential propagation number (R0) was 2.9 in a past report [30]. In Fig. (**4**), the schematic structure of SARS-Cov-2, as well as its potential diagnostic targets, are laid out for the reader [31]. Subsequently, treating outrageous Coronavirus cases is more unpredictable than treating SARS patients [32].

## 7 MECHANISM OF VIRAL INFECTIONS IN HUMAN

Coronavirus, which is one of the viruses that can cause the common cold, has been present in human populations for an extremely extended period of time. Due to the fact that the common cold is a contagious viral illness, the most common ways to catch it are by coughing, sneezing, and making contact with infected surfaces. Additionally, the disease can be spread through the inhalation or ingestion of virus droplets [33]. In the process of human coronavirus infection into susceptible host cells, the association between the viral S protein and cellular target receptors is a crucial early step that plays a significant role. Target receptor binding and specific recognition are carried out by S1, the S protein subunits. The membrane fusion is controlled by the other subunit, S2 [34]. Many human cells, particularly those in the lungs, have ACE2 receptors on their surface. The spike protein binds to these receptors to allow the virus to enter the body. The virus is ingested by human cells through a mechanism known as endocytosis. It has been proposed that COVID-19 most likely uses a special three-step mechanism for membrane fusion once it has entered the cytoplasm. This method requires the engagement of receptors and a change in the conformation of the spike glycoprotein, which is subsequently followed by the proteolysis of cathepsin L by intracellular proteases and further triggering the membrane fusion process within endosomes [35]. The endosome then breaks apart, allowing the virus to be released into the cytoplasm, and proteasomes, which are ordinarily able to hydrolyze proteins found in the host, are activated & start the process of uncoating the viral nucleocapsid (N) [36]. The S1 subunit of the virion binds to a receptor on the surface of the target host cell, and proteases on the host cell then break the Spike protein [37]. At last, the single-stranded RNA that makes up the viral genetic material is completely liberated into the cytoplasm. The processes of transcription and replication occur, which is known as the replication/transcription complex (RTC) [33].

## 8 IMMUNE RESPONSE AGAINST SARS-COV-2

Many specialists are right now investigating the safe situation's conceivable reaction to the infection. Most of them have effectively shown that during contamination, patients build up an unregulated, invulnerable reaction set off by macrophage and monocyte hyperactivation [38]. M immunoglobulins (IgM) are generally acknowledged as the principal line of assurance during viral diseases before the advancement of high partiality immunoglobulin G (IgG) for long-haul resistance and immunological cache. Notwithstanding, nitty-gritty proof of the human safety framework's reaction to SARS-CoV-2 contamination is still scant, and a lot of what is accessible is centred on what has been taken in over the course of the years from SARS-CoV & MERS-CoV diseases [39]. IgM can be distinguished in the infectant’s serum following 3–6 days after SARS-CoV contamination, while IgG could be recognized following 8 days [40]. On account of MERS-CoV contamination, sero-converting was found in the second or third week after the beginning of manifestations [41]. A postponed and helpless immunizer reaction was connected to outrageous results in the two types of COVID-19 contamination. Over the span of SARS-CoV-2 disease, a study [42] distinguished a potential invulnerable framework component. When recuperation blood tests were taken at four separate phases of the illness, IgM and IgG levels expanded consistently from day 7 to day 20, as indicated by the discoveries. The scientists found high convergences of cutting-edge T assistant cells (Th), Common Executioner cells (NK) & B cells in the blood test 7–9 days after the beginning of indications. SARS-CoV-2 contamination causes a resistant reaction similar to that seen during MERS-CoV contamination in a patient without attendant infections, as indicated by this report, and early versatile safe reactions might be connected to better clinical results [43].

## 9 IMMUNOLOGICAL BIOMARKERS

Biomarkers are crucial during this pandemic because they cause acceleration in the expansion as well as the establishment of advanced, novel therapies and medicinal commodities, especially vaccines [44]. Clinical biomarkers are biological measures of the occurrence, incidence, or form of the disease in medical settings that can be measured [45]. Biomarkers have gotten a lot of attention because they can be used to describe measurable features of a disease and to assess the best therapies based on these phenotypes and genotypes. Biomarkers associated with respiratory illness, for example, those related to intense respiratory trouble disorder (ARDS), have been connected to the expanded number of deaths (IL-8, ICAM-1) and upgraded endurance (IL-8, ICAM-1) (nitric oxide) [46]. Such molecular markers are significant in foreseeing possible entanglements or sickness seriousness, and they could be utilized to anticipate Coronavirus forecast [47].

A “cytokine storm,” or the widespread production of pro-inflammatory cytokines that lead to immediate lung injury and a poor prognosis, can be brought on by an infection with SARS-CoV-2 [48]. The virus is commonly detected in the lab by means of lymphocytopenia, neutrophilia, increased lactate dehydrogenase, CRP, D-dimer, Interleukins (IL)‒6, IL‒2 & IL-10 & decreased levels of natural killer (NK) and CD8^+^ T cells in particular [49, 50]. The different biomarkers used are detailed in Table **1**. The immunological response to SARS-CoV-2 infection may be responsible for tissue destruction in the kidneys, heart, liver, and lungs. A study on 1480 patients was done, and different parameters were evaluated. The most often assessed haematological indicators were platelet, neutrophil, WBC, and lymphocyte counts. Acute phase reactants included lactate dehydrogenase rate, erythrocyte sedimentation rate, D-dimer, CRP & procalcitonin levels. Total bilirubin, Gmma glutamyl transferase, Alanine aminotransferase (ALT), and Aspartate aminotransferase (AST) were measured in order to evaluate liver function. Creatine kinase, Troponin, myoglobin, and creatine kinase myocardial band levels served as indicators of cardiovascular disease. The gasometric parameters FiO_2_, pO_2_, pCO_2_, and pO_2_/FiO_2_, in addition to a chest x-ray, an evaluation of a computed tomography scan was conducted to assess lung participation and aid in the management of therapeutic decisions. The papers that were included in the study demonstrated that while CD8^+^ T cells seemed somewhat variable, total lymphocytes, CD3^+^ and CD4^+^ T cell counts were low during SARS-CoV-2 infection, particularly in severe and critical patients; white blood cells, neutrophil, platelet, and immunoglobulin levels were normal; and Tregs, fibrinogen, CRP, IL-6, TNF-α & ESR were increased regardless of the disease's severity [50].

Better prognostic prediction and the discovery of novel biomarkers and treatment approaches in the various stages of COVID-19 disease progression may be made possible by a more thorough compilation of data regarding the immunological & inflammatory timeline of SARS-CoV-2 infection [50].

Precision medicine based on systems biology approaches appears to be achievable with the development of biosensors that can gather time-course data from patients, liquid biopsies, and biomarker identification. The foundational elements for prediction models that employ systems biology techniques are supplied by these data collection instruments. Biosensors have the capability to gather and store quantitative data on physiological signals, such as heart rate and heart and brain electrical activity, and they can record real-time information on the concentration of various components in a patient's blood. Machine learning can be used to create a precise diagnostic tool for predicting models of disease states & progression due to advances in high-content picture analysis, which needs quantitative analysis of large quantities of images, such as pathology slides [51].

## 10 MUTATIONS IN SARS-COV-2 GENOME

### 10.1 Spike Mutations

Spike protein facilitates viral entrance by mediating virus coupling at the plasmalemma angiotensin-converting enzyme 2 (ACE2) receptor [25, 58, 59]. It is divided into two parts: S1 and S2. The receptor-binding domain (RBD) of the S1 unit can be directly connected with the ACE2 receptor [53, 60, 61]. The E484K mutation, which replaces glutamic acid E with lysine K, is also found in an originating variety derivational of B.1.1.7. Other than N501Y, both Beta and Gamma versions feature more substitutions [62, 63]. The S1 mutations increase ACE2 binding affinity while decreasing neutralizing antibody affinity, indicating a plausible explanation for their increased transmissibility and virulence. Since early 2020, another non-RBD mutation known as D614G has been carried by over 99 percent of prevalent variations [12, 58, 60].

### 10.2 NSP Mutations

NSP1 of ORF1a/ORF1ab and ORF8, two mutation hotspots, appeared to be linked with virulence and transmutable. The protein NSP1 helps the virus replicate by inhibiting type I interferon activation in the host. ORF8 is an immune-invasive protein that causes host cells to downregulate MHC-I. An early stop codon situated at position 27^th^ of ORF8 [64] was recently discovered in the Alpha form, which was discovered in a single immuno-compromised individual. NSP1 and ORF8 variants with partial deletions have been discovered. Though shortened NSP1 along with ORF8 proffer dainty infection and value a minimum number of infections globally (around 5%), it has emerged as one of the most common mutants in Africa in 2020 [58].

### 10.3 Alpha Variant

One of the first modified strains from the original SARSCOV-2 is the SARS-COV-2 Alpha variant. In September 2020, this variation was discovered in the United Kingdom. Several RBD mutations, including N501Y and P681H at positions 69–70 & 144 NTD deletions, as well as several non-spike mutations, were the main mutations generated by the Alpha variant, making this variant a variant of concern [65].

### 10.4 Beta Variant

The Beta variant was initially discovered in South Africa in May 2020. The beta variation exhibited several notable mutations that render it a variant of concern. These include nine spike protein changes, including RBD mutations, *i.e*., N501Y, E484K, and K417N & NTD deletions at positions 242-244 [66].

### 10.5 Gamma Variant

The gamma variant was initially discovered in January 2021 in Brazil. The gamma form of the virus includes around 22 mutations overall, with over 12 of those mutations occurring in the spike protein. L18F, N501Y, E484K, and K 417T are examples of RBD mutations. The gamma variant is shown to have NTD mutations as well. Compared to previously identified variants, this particular variant had a three to four times greater rate of hospitalization & morbidity [67, 68].

### 10.6 Omicron Variant

The Technical Advisory Group on SARS-CoV-2 Virus Evolution (TAG-VE) is an authoritative team of professionals who track as well as study SARS-evolution CoV-2's regularly, determining whether certain alterations or sets of variations affect the virus's characteristics. On November 26, 2021, the TAG-VE was summoned to evaluate the SARS-CoV-2 variant B.1.1.529 [69, 70].

On November 24, 2021, South Africa was the very first country to notify the transmission of the B.1.1.529 variant to WHO [71]. In South Africa, the epidemic scenario was already marked by three different spikes in confirmed instances; the Delta strain predominated in one of the most recent of these outbreaks. It has been stated that the rate of transmission has been steadily increasing over the past several days, which coincides with the finding of the B.1.1.529 strain [72]. A swab taken on November 9, 2021, has been the very first reported verified B.1.1.529 outbreak [73].

There are numerous variations in this genotype, including some that are problematic. In comparison to certain other VOCs, early research shows that, somehow, this variation has a greater chance of contracting the disease. In nearly all of South Africa's divisions, the occurrence of this variation escalated rapidly. This variation is still identified by conventional SARS-CoV-2 PCR investigations [74]. Numerous institutes recently reported that one of the three gene encodings is still not found through the extensively utilized PCR test, that such a test can thus have been employed as an indicator for such a mutant awaiting genomic validation [75]. A variety of investigations are now being conducted, and the TAG-VE will proceed to assess the modified version [76]. Depending on discoveries, WHO will inform the Member States and the general public about updates. The TAG-VE has informed the WHO that somehow, the recent mutation should be declared as a variant of concern (VOC), and the WHO has declared B.1.1.529 as a VOC, called Omicron, based on the information submitted that indicates a deleterious shift in COVID-19 demography [77, 78]. As a result, nations are being instructed to:

- Increase monitoring as well as testing attempts to understand the spreading of SARS-CoV-2 genotypes.
- Update complete genomic fragments and annotations to readily accessible platforms like GISAID.
- Use the IHR method to notify WHO about the first cases/clusters of VOC infection.

### 10.7 Covid 19 Variant XE

The WHO has defined three prototypes as of now, the first is Covid-19 Variant XD, the other one is Covid-19 Variant XF, and the third is Covid-19 Variant XE. More than 500 patients have been associated with it so far, according to WHO survey data; however, it has compelled a closer advantage of newer types. The WHO has stated that perhaps the COVID-19 Subtype XE variant will be extremely contagious than previous COVID configurations, but there is a lack of affirmation to substantiate this and demonstrate that this could legitimately produce another spike [79-81].

The new variation is a Covid-19 Variant XE cross between two Omicron variants: BA.1 and BA.2. As of now, it only accounts for a short fraction of cases globally. India’s cumulative COVID-19 data reached 43,027,035, while active cases are 13,445.

Figures that were updated at eight in the morning indicated that the death toll had increased to 5, 21, 264, including 83 additional deaths being recorded. Active cases correspond to 0.03 percent of total infections, according to the ministry, whereas the national COVID-19 rate of recovery remained at 98.76 percent. In a research that was published earlier this week, the World Health Organisation (WHO) noted, “The Covid-19 Variant XE recombinant, *i.e*., BA.1-BA.2 was first found in the UK on January 19, and less than 600 sequences have been reported and verified subsequently” [82].

### 10.8 JN.1 Variant

Every virus has distinct “spike proteins” that allow it to enter cells and produce specific symptoms. A new “variant” of the virus has emerged as a result of additional modifications, or “Mutations,” in the DNA sequence of those spikes. Variants may vary in how severe they are, how easily they spread, and how they will respond to various symptom therapies. The most recent mutation, known as BA 2.86, gave rise to the novel coronavirus strain. JN.1. The latter belongs to the same family as the more severe COVID-19 strain known as the “Omicron” variant, which peaked last year. The latest variation shows more genetic differences from its predecessors, indicating that the virus is still evolving [83].

## 11 CLINICAL IMPLICATIONS OF SARS-COV-2

Elevated viral virulence as well as transmissibility due to a stronger affinity for olfactory epithelium, the S-protein mutation D614G has been demonstrated to affect SARS-CoV-2 transmissibility rate & it has been demonstrated to acquire more transmutability, particularly in animal models [84]. The VOCs Alpha and Beta have been shown to improve transmutability by 50%, particularly in younger age groups and youngsters [62, 85]. Alpha variants have been linked to an increase in hospitalizations and mortality, which may be owing to their ability to evade neutralizing Abs due to RBD mutations [58].

### 11.1 Reduced Diagnostic Susceptibility

The novel VOCs may impair the receptiveness of RT-PCR-centric test techniques, particularly in a case where alteration takes place near primers and probes [85]. According to reports, at least one genome has already mutated 79 percent of the binding locations for primers employed in the RT-PCR experiment, with the GGG AAC mutation having the most relevance [15]. In the global SARS-CoV-2 genomes, a recent study found that binding locations for mapped primers or probes found an aggregate variation prevalence attributed to 1% [61]. A supplementary source of worry came out to be the S deletion mutant (H69-V70), which was discovered in France and has been linked to difficulty in capturing S-gene expression in three-target RT-PCR [86]. Other investigations found that dissimilarities within probe bonding areas of SARS-CoV-2 test evaluation might be discovered in distinct SARS-CoV-2 mutants [87].

## 12 POSSIBLE WAY TO THE EXISTING VACCINATIONS

### 12.1 mRNA Vaccines

BNT162b2 (Pfizer-BioNTech) & mRNA-1273 (Pfizer-BioNTech) are two key mRNA-based anti-SARS-CoV-2 vaccines that have been accepted (Moderna). According to studies, BNT162b2 therapy produces potential antibodies for neutralizing Alpha and Gamma mutants with a slightly lesser potential with respect to the Beta variant [58].

### 12.2 Adenovirus-based Vaccines

For general or emergency use, four adenovirus-based vaccinations have been approved. The chimpanzee adenovirus-vectored vaccination ChAdOx1 Covid-19 (Oxford) expresses the SARS-CoV-2 spike protein. According to recent investigations, ChAdOx1 Covid-19 was 74.6 percent effective against Alpha but just 10.4 percent effective against Beta [88]. There is no information available about its effectiveness in reducing VOCs [89].

### 12.3 Inactivated Virus-based Vaccines

Three vaccines based on inactivated viruses have been licensed so far for use in countries like China, India, as well as Brazil. According to the novel *in vitro* investigation, antisera extorted through the BBIBP-CorV vaccine (Sinopharm) can neutralize the Beta variant at a lesser level than the wild- type strain and the D614G variant. BBV152 (Bharat Biotech International Limited) vaccinated human serum can neutralize the Alpha variant, according to a recent serological investigation [58]. The vaccination is a potential counter to the Alpha and Gamma versions, according to the same research centre [90].

## 13 EXISTING TREATMENT OPTIONS FOR THE MANAGEMENT OF COVID 19

Scientific groups are under pressure to discover novel medicines quickly in response to the COVID-19 outbreak. Moreover, a significant advancement in pandemic management is the length of time needed for the development, synthesis & assessment of novel medications from preclinical to phase III trials. Two main pathways are thought to be responsible for COVID-19 pathogenesis. SARS-CoV-2 replication is the primary source of disease in the early stages of clinical manifestation. Tissue damage resulting from an immunological or inflammatory response that is dysregulated in response to SARS-CoV-2 seems to be the primary cause in the late clinical phase. It is anticipated that treatment aimed at precisely targeting SARS-CoV-2 will be most effective in the early stages of the illness, whereas immune-suppressive or anti-inflammatory medications will likely be more beneficial in the later stages of COVID-19 [91].

### 13.1 Antiviral Drugs

Remdesivir is one of the most promising drugs and the only one approved by the FDA for treating COVID-19 patients. Remdesivir was first used to treat Ebola virus infection. It is a phosphoramidite prodrug of adenosine nucleoside with broad antiviral action [92]. Remdesivir binds to the viral RNA-dependent RNA polymerase and prematurely ends RNA transcription, hence inhibiting viral replication [93]. Patients undergoing solid organ transplantation may benefit from combination therapy that includes the immunomodulatory medication tocilizumab and the Remdesivir medicine for COVID-19 treatment [94].

Presently, the FDA has approved two new antiviral medications under the brand names Paxlovid & Molnupiravir for oral use in COVID-19 patients. The first oral medication approved by the FDA under an emergency use authorization for the treatment of COVID-19 instances is called Paxlovid. Another FDA-approved oral medication under the emergency use authorization for COVID-19 patients' therapy is Molnupiravir [91]. A number of drugs, including licensed antimalarials like Chloroquine & anti-protozoan drugs like ivermectin and emetine, have also been explored for SARS-CoV-2 infection [95]. These drugs exhibit antiviral or immunomodulatory properties *in vitro*. Nonetheless, due to its high toxicity and dubious benefits, the Infectious Diseases Society of America & NIH panels strongly advised against using Chloroquine either alone or in conjunction with Azithromycin [91, 96].

### 13.2 Plasma Therapy

The FDA trusted Source revised its guidelines on the use of convalescent plasma in February 2021. It also claimed that convalescent plasma must pass a high titer (high concentration) test before being used. A promising strategy for stopping viral reproduction with a high chance of total viral clearance is the high level of antibodies in convalescent patient plasma that has been observed in several viral infections [97]. The Convalescent plasma from coronavirus-infected patients was therefore suggested to be useful in reducing severe symptoms in people experiencing serious problems [98]. Several studies in an extensive group of hospitalized COVID-19 patients showed that receiving convalescent plasma decreased mortality and the requirement for artificial respiration [91].

### 13.3 Anti-inflammatory & Immunomodulatory Agents

#### 13.3.1 Corticosteroids

Moreover, because many corticosteroids have strong anti-inflammatory properties, they are utilized to lower inflammation in a variety of autoimmune disorders. Corticosteroids were previously used in patients with severe SARS infection; however, due to the severe effects of COVID-19 infection on the immune system, notably in cytokine dysregulation, they are now included in COVID-19 therapy regimens [99]. Methylprednisolone and dexamethasone, two powerful and often used corticosteroid medications for lung treatment, were included in the COVID-19 standard treatment regimens [91].

Ranjbar *et al*. examined the effectiveness of methylprednisolone & Dexamethasone in treating 86 hospitalized COVID-19 patients who were hypoxic. The patients were divided into three groups: one receiving methylprednisolone treatment (2 mg/kg/day), the other receiving Dexamethasone treatment (6 mg/day), and the control group receiving neither treatment. The two groups receiving intravenous corticosteroid treatment did not significantly differ from one another, according to the data, while methylprednisolone was somewhat more potent than dexamethasone. However, after ten days of administration of corticosteroids, there were notable improvements in the clinical status of the corticosteroid-treated groups compared to the control group, as well as a notable decrease in hospitalization duration and ventilation reliance [91, 100].

#### 13.3.2 Interleukin-6 Inhibitors

The anti-IL-6 receptor monoclonal antibodies (mAbs) Sarilumab and Tocilizumab, as well as the anti-IL-6 mAb Siltuximab, are approved by the FDA as interleukin-6 (IL-6) inhibitors. In patients with severe COVID-19, tocilizumab, an immunomodulatory monoclonal antibody, showed encouraging therapeutic results. Since a high titer of IL-6 is typically linked to severe COVID-19 infection, tocilizumab's mode of action is based on its capacity to inhibit IL-6 receptors (175). A single-centre trial with 100 hospitalized patients showed that tocilizumab intravenous injection significantly improved clinical outcomes in the treated group as compared to the control group [91, 101].

### 13.4 Lactoferrin

Natural glycoprotein lactoferrin has a variety of biological properties, such as immunomodulatory, antioxidant, and antiviral properties [102]. Lactoferrin's ability to impede the entry of viruses into host cells by quenching their particles or blocking their receptor has been demonstrated in multiple investigations, demonstrating its effectiveness against a wide range of viral infections [103]. Numerous coronaviruses have been found to use this pathway, which involves inhibiting the HSPG surface protein *in vitro*-a crucial co-receptor for COVID-19 entrance [104]. Furthermore, lactoferrin demonstrated noteworthy immunomodulatory and anti-inflammatory properties, which could account for its incorporation into certain COVID-19 treatment protocols [91, 105].

### 13.5 Adjuvant Therapy Used in COVID-19 Patients

The mortality risk identified in hospitalized patients is thromboembolism after acute pneumonia from a severe COVID-19 infection [106]. Numerous research investigations have documented a rise in the prevalence of coagulation markers, such as fibrinogen, D-dimer, and extended thrombin time, following viral infection [107]. In COVID-19 patients, heparin, a strong and widely used anticoagulant, is the drug of choice. Heparin exhibits anti-inflammatory, antiviral, and anticoagulant properties [108]. Numerous investigations have demonstrated that the administration of heparin to COVID-19 patients produced noteworthy therapeutic benefits without any negative side effects [109].

Improving the immune system is a prime target and cure for COVID-19 prophylaxes (202) [110]. Common supplements for treating viral influenza include zinc and vitamin C, which help the immune system fight the infection more effectively. Zinc may have immunomodulatory and antibody-inducing properties, while Ascorbic acid (vitamin C) is a well-known antioxidant [91, 111].

### 13.6 Herbal Medicines

The root glycyrrhizin was found to interfere with SARS-CoV reproduction in the lab. Isoflavones in Lambda Scutariiária have been shown to have bacterial biomass against COVID-19 in the laboratory. Polysaccharide protease, derived first from the wood of the Alnica indica genus, was found to be impotent against the proteolytic enzymes from infections. As a result, herbal medicines can be used to improve COVID-19 immunity (Table **2**) [112, 113].

## 14 VACCINE

Nucleic corrosive antibodies (counting mRNA and DNA immunizations), recombinant hereditary designing (protein recombinant) immunizations, inactivated immunizations, weakened flu infection vector immunizations, and adenovirus vector immunizations are among the forthcoming Coronavirus immunizations [114, 115]. The SARS and bird flu candidates use cytotoxic T-lymph epitopes and B-lymph antigens to mount a second immune response [116]. A few analysts propose that antibodies could be created utilizing the whole S protein or the S1 protein containing the RBD [39]. Immunizations focusing on antibodies against S2 straight epitopes, then again, could be more powerful than immunizations focusing on antibodies against S1 subunit because there are fewer hereditary bungles that make SARS-CoV-inferred antibodies inadequate, as indicated by certain reports [117]. Propelling investigation of option COVID antibodies needs more grounded worldwide collaboration [116, 118, 119].

### 14.1 Potential COVID-19 Vaccines

#### 14.1.1 Live Attenuated Vaccines (LAVs) and Inactivated Vaccines (IVs)

LAV is a virus vaccination that is alive yet non-virulent. Furthermore, because of their long-term experience, LAVs have a strong chance of being the first vaccine candidate for the COVID-19 pandemic. They do, however, have several drawbacks, the most significant of which is the need for cold-chain dissemination. Three vaccines based on inactivated viruses have been licensed so far for use in countries like China, India, as well as Brazil. The vaccination is a potential counter to the Alpha and Gamma versions, according to the same research centre [58].

#### 14.1.2 mRNA Vaccines

BNT162b2 (Pfizer-BioNTech) and mRNA-1273 (Pfizer-BioNTech) are two key mRNA-based anti-SARS-CoV-2 vaccines that have been accepted (Moderna). According to studies, BNT162b2 therapy produces potential antibodies for neutralizing Alpha and Gamma mutants with a slightly lesser potential with respect to the Beta variant [58].

#### 14.1.3 Next-generation Vaccines

Pathogens could be classified as a type of nanostructure. Nanotechnologies include LAVs, IVs, and viral vectors, to name a few. Nanotechnology-based vaccine development and immune-engineering techniques are particularly effective. The rationale for this is that viruses and nanoparticles both act on the same length scale. The structural properties of nanoparticles, including organic and manmade, are identical to those of pathogens. Chemical biology, biotechnology, and nanochemistry were used to develop next-generation designer vaccination technologies.

#### 14.1.4 Nucleic Acid-based

The Phase I clinical study for Inovio Pharmaceuticals and Entos Pharmaceuticals, Inc., both situated in Canada, began in April 2020. Moderna began phase I testing a new treatment about the RNA-installed method in the United States on March 16, 2020. Biotech-Pfizer recently announced that Phase I/II testing that was conducted in Germany has been approved to investigate four forefront mRNA vaccine successors. Insertional mutagenesis is not a concern with mRNA vaccines. Researchers are collaborating on a study to extend the RNA's short half-life and boost S protein expression levels. Cationic liposomes and polymeric nanoparticles are two examples of synthetic carriers.

#### 14.1.5 Peptide-based Vaccines

This is an immunotherapy strategy that involves administering a peptide with an immune-subservient to activate T-cell and B-cell immunity. In earlier investigations of the SARS-CoV and MERS-CoV vaccines, it has been proposed that elevated IgG seroprevalence is linked to poor results. Peptide-based vaccines are the most basic type of vaccination that can be efficiently created, validated, and made quickly [120]. Nonetheless, concerning clinical risks, such divisions are typically excluded from ongoing trials, causing sample size issues and lowering the quality of the created vaccine.

### 14.2 Current Status of COVID-19 Vaccines

A massive experiment with 43,548 participants from 152 sites around the world, the majority of which were in the United States, has just been published. There have been no fatalities linked to the vaccination. Most of the disadvantageous occurrences were described as transient unadjuvanted episodes. A phase 1 trial in younger and older individuals in the United States of two vaccine successors, BNT162b1 and BNT162b2, has been completed. There have been no fatalities linked to the vaccination. mRNA- 1273, a further mRNA therapy, however, has accomplished its phase I trial. They have progressed to a crucial phase 2–3 preclinical and clinical assessment [120].

Although phase 3 efficacy investigations have demonstrated the value of vaccines against COVID-19, they have not always thoroughly assessed the protection offered by prior infection and hybrid immunity. Based on the participants' prior infection status at enrollment and treatment, researchers divided them into four groups for this post-hoc cross-protocol evaluation of the Moderna, AstraZeneca, Janssen, and Novavax COVID-19 vaccine clinical trials: no prior infection/placebo, prior infection/placebo, prior infection/vaccine, and prior infection/vaccine. Those who had already been infected or received a placebo had a 92% lower chance of contracting COVID-19 in the future than those who had neither; for individuals who received a single dose of Janssen, hybrid immunity provided more protection than the vaccine alone. Prior to study enrollment, SARS-CoV-2 infection offered significant protection against reinfection in the blinded/pre-crossover monitoring of the four clinical trials. Nonetheless, the COVID-19 vaccination also guards against the dangerous side effects and potential for transmission of SARS-CoV-2 infection. Hybrid immunity might offer other advantages. Clinicians and public health professionals ought to urge everyone, irrespective of their infection history, to get vaccinated against SARS-CoV-2 [121].

The pandemic is a constant obstacle for patients with impaired immune systems. This diverse group, which includes people with HIV infection, cancer, transplants, primary immunodeficiencies, and those receiving treatment with immunosuppressive biologics and drugs, makes up roughly 2-3% of the total population. Some people (such as transplant recipients) may not respond well to vaccinations, which can increase their chance of contracting COVID-19 prolonged viral shedding and experiencing serious infection-related consequences. One of the problems with immunocompromised patients is that they shed the virus rather slowly. Immunocompromised patient populations frequently have weakened immune responses. Patients receiving solid organ transplants, those with hematologic malignancies, elderly patients, and those on corticosteroids, immunosuppressive medications, or anti-CD20 drugs should be especially concerned. Immunocompromised individuals require modified vaccination schedules since a primary series of two doses does not produce immunological responses comparable to those of non-immunocompromised individuals. For all immunocompromised patients, the WHO consequently advises an additional dosage, known as the “extended primary series.” Immunocompromised patients are one such category. It is necessary to conduct studies to learn more about the effectiveness of the antiviral drugs that are now on the market, both individually and in combination, and to examine their use when the disease progresses more slowly than in the general population [122].

Determination of the entire scenario of Covid-19 is necessary because SARS-CoV-2 sub-variations and variants continue to emerge following booster doses and immunizations. Suleiman *et al.* performed a secondary meta-analysis of co-infection, subsequent infections & antimicrobial resistance in COVID-19 patients. Co-infections contribute to a greater death rate for COVID-19 patients, with some of the worst outcomes occurring in severely ill patients. Following a COVID-19 infection, individuals may acquire subsequent bacterial infections that could be fatal. Bacterial co-infections caused the death of several COVID-19-positive patients who had no preexisting medical disorders, such as diabetes, hypertension, heart problems, kidney, or liver abnormalities. This may lead to a marked rise in the number of people who test positive for COVID-19 and die. As a result, researchers should pay more consideration to this matter. It is apparent that additional study is needed to establish the precise impact of co-infection and SARS-CoV-2 on clinical outcomes [123].

## CONCLUSION

Due to the extensive use of vaccinations and protection against natural illnesses, the COVID-19 pandemic has been controlled & daily infections have decreased globally. The advancement of treatment prospects against SARS-CoV-2 is primarily dependent on the findings discovered during the investigation of other coronavirus diseases. While certain medicines that show promise in the fight against COVID-19 have antiviral effectiveness against SARS-CoV-2 variants and other coronaviruses, not all of them are acceptable for all patients due to possible adverse effects. The function of spike S protein & RBD during SARS-CoV-2 infection in the production of protective immunity & humoral immunity *via* T-cell responses & neutralizing antibodies has led to the development of numerous novel vaccines and medications. Future research on SARS-CoV-2 is crucial to provide vital information for creating and developing novel vaccines as well as therapy and prophylactic measures against the newly evolving variants of this virus, considering the high rates of disease and death associated with COVID-19. Therefore, in order to ensure that the recently developed vaccines are safe and suitable for use in humans, it is imperative that they be validated using animal models. Reducing rates of morbidity and mortality could result from understanding the mechanism of COVID-19 infection and strengthening the immune system.

Researchers can work more quickly and accurately on SARS-CoV-2 studies, epidemiological analyses, drug impact simulations, and vaccination development by utilizing computer systems like artificial intelligence. Effective domains can be identified to speed up the modification of current effective medications and generate broad-spectrum, targeted therapies against coronaviruses by simulating infection mechanisms, screening vast drug libraries using models and algorithms, and forecasting potential mutation types.

## Figures and Tables

**Fig. (1):**
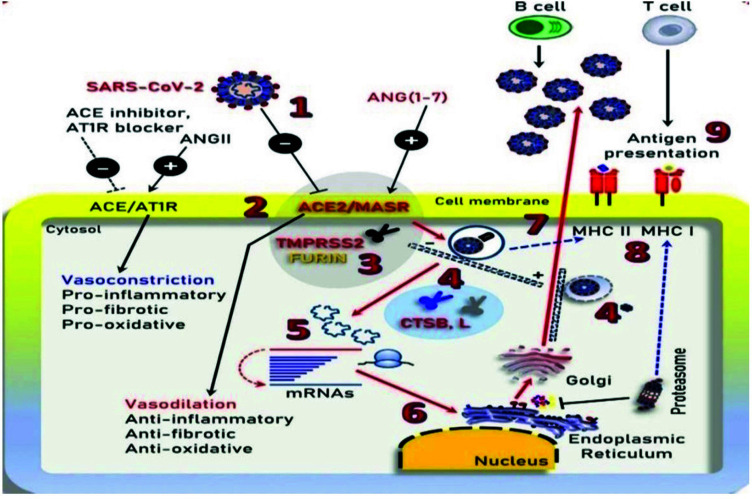
Illustration of the main cell signalling axes. **1**- SARS-CoV-2; **2**- binding to Angiotensin Converting Enzyme-2 receptor; **3**- FURIN priming; **4**- Endocytosis mediated by clathrin; 4*- compartments of endosomes during exocytosis; **5** and **6**- Uncoating, release of RNA and synthesis of viral-protein in both endoplasmic-attached and unattached ribosomes; **7**- Exocytosis mediated by vesicles; **8**- Antigen presentation by Major Histocompatibility Complex I and II; **9**- Adaptation of immunity and infected cells’ elimination [5].

**Fig. (2):**
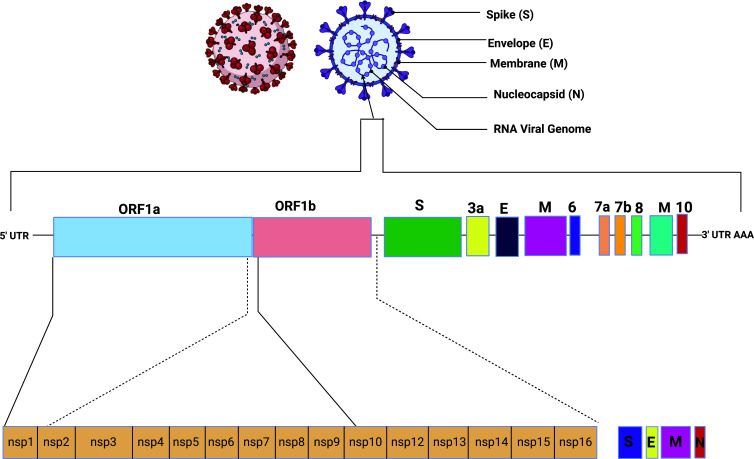
The genomic materials of SARS-CoV-2 [11].

**Fig. (3):**
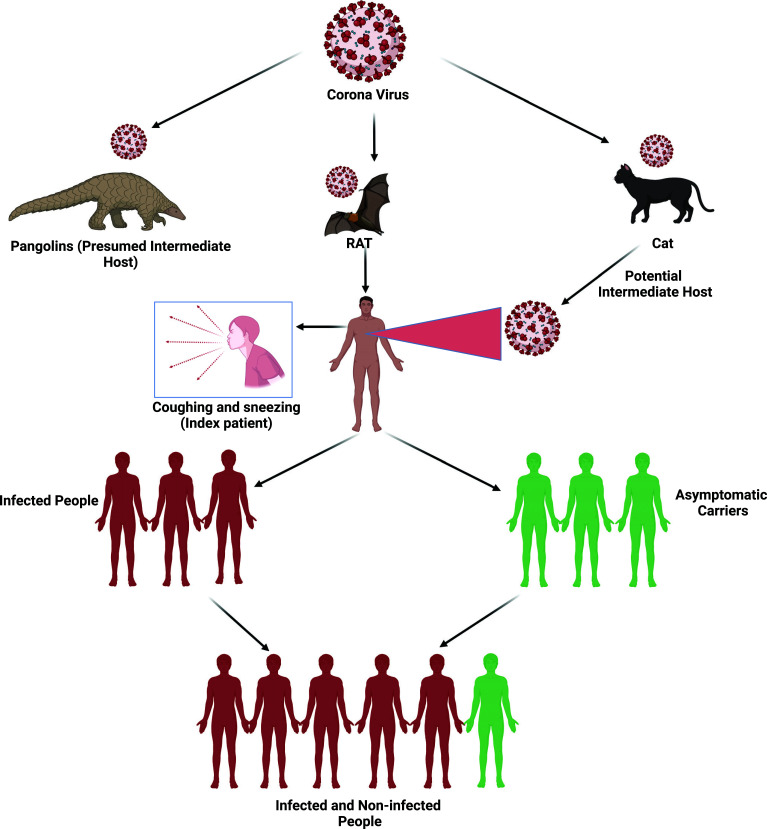
Transmission channel of SARS-COV-2 [24].

**Fig. (4):**
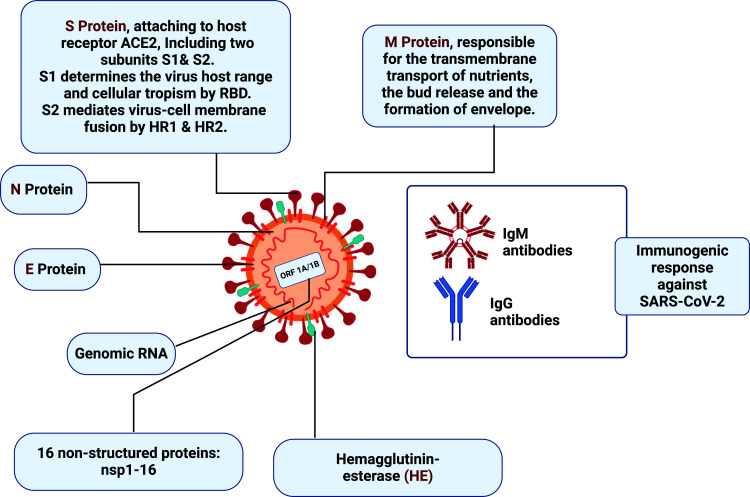
Schematic structure of SARS-CoV-2 and its possible targets for diagnosing [31].

**Table 1:** Biomarkers and their descriptions.

**Biomarkers**	**Descriptions**
Clinical biomarkers	Recently, a method for identifying serious cases among COVID-19 patients that is routinely used in hospitals has been introduced, reviewed, and analyzed [52]. Important variations in serum direct cholinesterase, bilirubin & concentration of lactate dehydrogenase (p 0.05) have also been identified between moderate and mild COVID-19 patients. As a consequence of this, determining the severity of the disease by the use of these serum markers is essential for bringing the death rate down.
Inflammatory	They have likewise been connected to the reality of the illness. The levels of all incendiary markers estimated were essentially higher in genuine patients contrasted with non-genuine cases in a methodical report [53]. Moreover, it affirms that higher IL-6 levels are connected to higher death rates. PCT esteems likewise connected to an almost 5-crease expanded danger of being outrageous. This threat is supposed to be expanded in outrageous Coronavirus cases [54].
Immune defending cells	They are a portion of the primary human cells to respond to the SARS-CoV-2 assault. In a new report, each of the 20 Coronavirus patient members created CD4 Lymphocytes & antibodies IgG and IgA focusing on the viral S-protein, with 70% of cases delivering discernible CD8 Immune system microorganisms [55]. These discoveries show that the human invulnerable framework is fit for mounting a huge and durable reaction to the novel COVID-19. Lymphopenia has additionally been found in Coronavirus patients, with lower levels of CD4 and CD8 immune system microorganisms in outrageous cases contrasted with moderate cases, albeit these levels were reestablished once the viral contamination was taken out [6, 56].
Age	Coronavirus seriousness is altogether affected by age. Nonetheless, previous elements (counting diabetes, coronary illness, kidney infection, hypertension, overweight, and way of life) are identified with a higher danger of death. This wonder recommends that for Coronavirus, organic age (the presence of one's body) is similarly pretty much as fundamental as sequential age (the number of living years). Organic age is controlled by hereditary qualities, way of life propensities, medical issues, and harmful openings (microorganisms or synthetics). The epigenetic clock & the glycan clock are two of the most compelling biomarkers of the natural periods. Since IgG glycosylation is related to both ordered and natural ages, glycan timekeepers, which are associated with an unfortunate way of life markers, might be valuable apparatuses for biomarker disclosure [57].

**Table 2:** Clinical trial updates on Ayurveda, extract, and herbal medicine for the management of COVID-19. (https://clinicaltrials.gov/).

**S. No.**	**Title of Trial**	**Phase**	**Status**	**Conditions**	**NCT Number**	**Location**
1.	Ayurveda as preventive therapy for people suspected of having COVID-19.	Not applicable	No recruitment yet	COVID-19	NCT04395976	United Kingdom
2.	Traditional Chinese medicines for the treatment of severe COVID-19.	Phase III	No recruitment yet	COVID-19	NCT04323332	China
3.	Prevention & treatment of COVID-19 using traditional Chinese medicines.	Not applicable	Recruiting	Human Coronavirus causing Pneumonia	NCT04251871	China
4.	Patients with rheumatoid arthritis who take anti-rheumatic medications show an increased chance of contracting the COVID-19 infection.	-	Enrolling by invitation	➢Rheumatoid arthritis ➢COVID-19	NCT04434118	Egypt
5.	Yinhu Qingwen decoction for treating moderate COVID-19.	Phase II Phase III	Suspended	➢COVID-19 ➢Chinese medicine	NCT04278963	China
6.	COVID distinction in accordance with traditional Chinese medicines, as well as a treatment procedure.	-	Completed	COVID-19	NCT04306497	China
7.	YinhuQingwengranula to mitigate the effects of severe COVID-19.	Phase II, Phase III	Suspended	➢COVID-19 ➢Severe Pneumonia ➢Chinese medicines	NCT04310865	China
8.	A randomized controlled experiment to investigate the effectiveness of Lianhua Qingwen as adjuvant therapy in individuals who were reported with moderate symptoms of COVID-19.	Phase III	No recruitment yet	COVID 19	NCT04433013	-
9.	A description of telehealthcare based on Chinese herbal medicine for the treatment of symptoms associated with viral illnesses such as COVID-19.	-	Recruiting	SARS-CoV-2 infection	NCT04380870	United States
10.	Hesperidin & Diosmin as a potential therapy for COVID-19.	Early phase I	No recruitment yet	Coronavirus infection	NCT04452799	-
11.	A survey on individual experiences with the traditional Chinese medication Jing-Guan-Fang for the treatment and prevention of COVID-19.	-	Recruiting	Coronavirus infection	NCT04388644	Taiwan
12.	The trial against COVID-19 using Honey & *Nigella sativa*.	Phase III	Recruiting	SARS-CoV-2 infection	NCT04347382	Pakistan
13.	Colchicine and phenolic monoterpenes have been shown to be effective in treating COVID-19.	Phase I Phase II	Recruiting	COVID 19	NCT04392141	Iran
14.	Potential use of gum Arabic as an immune-modulating drug in individuals diagnosed with COVID-19.	Phase II Phase III	No recruitment yet	COVID 19	NCT04381871	Sudan
15.	Colchicine in moderate symptomatic COVID-19 patients.	Not applicable	Recruiting (new)	COVID 19	NCT04527562	Bangladesh
16.	Evaluation of the effectiveness & safety of Guduchi Ghan Vati in asymptomatic COVID-19 patients.	-	Completed	COVID 19 asymptomatic condition	NCT04480398	India
17.	Self-management using Ayurvedic techniques for flu- like symptoms throughout the COVID-19 epidemic.	Not applicable	Completed	COVID 19 flu-like symptom flu-like illness	NCT04345549	United Kingdom
18.	Ayurveda treatment for flu-like symptoms during the COVID-19 epidemic.	Not applicable	Completed	COVID-19 flu-like illness	NCT04351542	United Kingdom
19.	Providing protection to healthcare workers during the COVID-19 epidemic.	-	Completed	COVID-19	NCT04387643	India
20.	The effect of combining therapy with the capsules of reginmune and the tablets of immuno free on the management of COVID-19 in patients with mild to moderate symptoms.	Phase II Phase III	Recruiting (new)	Treatment of Covid-19 virus infection	NCT04494204	India
21.	Lessons learnt from COVID-19: The goal of the TOMEKA^®^ campaign is to educate.	-	No recruitment yet	COVID-19	NCT04424940	Congo
22.	In asymptomatic COVID-19 patients, the use of phytomedicines as an additional treatment to Azithromycin compared with the use of hydroxychloroquine.	Phase II	Enrolling by invitation (new)	COVID-19	NCT04501965	Guinea

## LIST OF ABBREVIATIONS

ACE2: Angiotensin-converting Enzyme 2
ARDS: Acute Respiratory Distress Syndrome
GISAID: Global Initiative on Sharing Avian Influenza Data
MERS: Middle East Respiratory Syndrome
MHV: Murine Hepatitis Infection
NTD: N-terminal Domain
ORFs: Open Reading Frames
RBD: Receptor-binding Domain
SARS-CoV: Severe Acute Respiratory Syndrome Coronavirus
VOC: Variants of Concern
